# Polypharmacy Prevalence Among Older Adults Based on the Survey of Health, Ageing and Retirement in Europe: An Update

**DOI:** 10.3390/jcm14041330

**Published:** 2025-02-17

**Authors:** Elena Gatt Bonanno, Teodora Figueiredo, Inês Figueiroa Mimoso, Maria Inês Morgado, Joana Carrilho, Luís Midão, Elísio Costa

**Affiliations:** 1 Faculty of Medicine and Surgery, University of Malta, 2090 Msida, Malta; 2 CINTESIS@RISE, Biochemistry Lab, Faculty of Pharmacy, University of Porto, 4099-002 Porto, Portugal; 3 Department of Biological Sciences, Faculty of Pharmacy, University of Porto, 4099-002 Porto, Portugal; 4 Porto4Ageing—Competences Centre on Active and Healthy Ageing, Faculty of Pharmacy, University of Porto, 4099-002 Porto, Portugal

**Keywords:** polypharmacy, prevalence, SHARE, aging, older adults

## Abstract

Polypharmacy, a common condition among the older population, is associated with adverse outcomes, including higher mortality, falls and hospitalization rates, adverse drug reactions, drug–drug interactions, medication nonadherence, and consequently increased healthcare costs. **Background/Objectives**: This study aims to explore the prevalence of polypharmacy and its associated factors among older adults across 27 European countries and Israel. **Methods**: In this cross-sectional analysis, we used data from participants aged 65 years or older from Wave 9 of the Survey of Health, Aging, and Retirement in Europe (SHARE) database. The variables studied were classified into the following categories: sociodemographic, behavioral factors, physical functioning, physical health, mental health, and living conditions. **Results**: Our results showed an overall prevalence of polypharmacy of 36.2%, ranging from 25.0 to 51.8%. Slovenia, Greece, and Switzerland were the countries with the lowest prevalence, whereas Portugal, Israel, and Poland were the countries where the prevalence of polypharmacy was the highest. Polypharmacy was shown to be associated with variables from all categories. **Conclusions**: Polypharmacy is a highly prevalent condition in the older population. Identification of variables associated with polypharmacy, such as those identified in this study, is important to identify and monitor older groups, which are most vulnerable to polypharmacy. Interventions designed to reduce polypharmacy should consider these associations.

## 1. Introduction

Polypharmacy, the simultaneous use of multiple medications, is a growing global concern, particularly among the older population [1]. Although there is no universal consensus on the specific threshold for defining polypharmacy, the definition by Bjerrum et al., which describes it as the concurrent use of five or more daily medications, is widely accepted [2]. While the term typically pertains to prescription drugs, it also encompasses over-the-counter medications, dietary supplements, and other health-related products [3,4].

The rise in polypharmacy is often attributed to increased life expectancy, which is accompanied by a higher prevalence of chronic diseases. This situation implies the use of multiple medications to manage symptoms and mitigate complications arising from multimorbidity [5]. Advanced age is commonly associated with physiological changes such as altered pharmacokinetics and pharmacodynamics, reduced liver and kidney function, diminished muscle mass, compromised flexibility of the immune system, poor nutritional status, and lower hydration levels. These factors contribute to the fact that nearly 40% of individuals aged 65 and older have two or more chronic conditions, with prevalence increasing as age advances [6,7,8]. In addition, a Danish study found that approximately 53% of individuals aged 60 and older used medications from over five distinct drug classes [9], a trend mirrored in various other countries [10].

Polypharmacy, or the use of multiple medications, is well-known to carry associated health risks. These risks can lead to various adverse health outcomes, which can be categorized into five main areas: patient-related consequences, challenges faced by healthcare professionals, burdens on caregivers, impacts on the healthcare system, and economic repercussions [4,11,12]. Additionally, inappropriate self-medication and the misuse of over-the-counter drugs further complicate these issues [13]. A retrospective cohort study revealed that polypharmacy is linked to a higher risk of being prescribed potentially inappropriate medications, as well as an increase in outpatient visits and hospitalizations, resulting in an approximately 30% rise in medical costs [14]. Furthermore, in a prospective cohort study involving older hospitalized adults taking five or more medications, a staggering 80% prevalence of potential drug–drug interactions mediated by the hepatic cytochrome enzyme was observed [15]. A population-based study also indicated that outpatients on five or more medications faced an 88% greater risk of experiencing an adverse drug event (ADE) compared to those taking fewer medications [16].

While polypharmacy is often regarded as a clearly defined and uniform phenomenon, emerging evidence suggests that its prevalence and characteristics can vary significantly across countries [17]. In a previous study, carried out in 2018, the prevalence of polypharmacy in older adults was evaluated in 17 European countries and Israel. It was found that the prevalence of polypharmacy was lower in Switzerland (26.3%), Croatia (27.3%), and Slovenia (28.1%), and higher in Portugal (36.9%), Israel (37.5%), and the Czech Republic (39.9%) [18]. This study aims to explore the prevalence of polypharmacy and its associated factors among older adults across 27 European countries and Israel. The analysis utilizes data from wave 9 of the SHARE (Survey of Health, Aging, and Retirement in Europe) project, a robust international database providing detailed insights into the demographics, health, and socioeconomic conditions of individuals from community-dwelling populations. This study aims to analyze an association between polypharmacy and sociodemographic factors, behavioral factors, physical functioning, physical health, mental health, and living conditions, respectively.

## 2. Materials and Methods

This study exploits data from Wave 9 of the SHARE (Survey of Health, Aging, and Retirement in Europe) project (https://doi.org/10.6103/SHARE.w9.900). SHARE is an international and interdisciplinary database that captures detailed information on the health, social and economic conditions, as well as the family and social networks, of representative community-dwelling populations. The dataset includes participants from 27 European countries (Austria, Belgium, Bulgaria, Croatia, Cyprus, the Czech Republic, Denmark, Estonia, Finland, France, Germany, Greece, Hungary, Italy, Latvia, Lithuania, Luxembourg, Malta, the Netherlands, Poland, Portugal, Romania, Slovakia, Slovenia, Spain, Sweden, and Switzerland) in addition to Israel. Recognized as a cornerstone of aging research in Europe, SHARE provides invaluable insights into the complexities of aging. Wave 9 includes data from 69,447 individuals, ranging in age from 20 to 106 years.

### 2.1. Prevalence of Polypharmacy

To assess the prevalence of polypharmacy, we selected all individuals aged 65 years or older from Wave 9 of the SHARE project who responded to the question: “Do you take at least five different drugs on a typical day? Please include drugs prescribed by your doctor, drugs you buy without a prescription, and dietary supplements such as vitamins and minerals”. For this study, polypharmacy was defined, as previously mentioned, as the simultaneous use of five or more medications per day.

### 2.2. Explanatory Variables

SHARE encompasses a comprehensive range of data spanning multiple domains, enabling the analysis of numerous exploratory variables. These variables were organized into six major categories: sociodemographic, behavioral factors, physical functioning, physical health, mental health, and living conditions.

#### 2.2.1. Sociodemographic Variables

This category included gender, age, marital status, education level, and shortage of money. Gender was classified as male or female. Age was calculated based on the participants’ reported year of birth relative to 2022 and grouped into three categories: 65–74 years, 75–84 years, and 85+ years. Marital status was assessed based on self-reported responses and categorized accordingly. Education level was determined by the total number of years spent in full-time education and grouped into four levels. Shortage of money was evaluated using a question about financial constraints affecting daily activities.

#### 2.2.2. Behavioral Factors

This category included smoking history, alcohol intake, physical activity, and dietary habits. Ever smoking daily was assessed based on whether participants had smoked any form of tobacco for at least one year. Alcohol intake was evaluated based on self-reported consumption in the last seven days. Physical activity was assessed through two separate variables: vigorous physical activity and moderate physical activity, with frequency categorized into four levels. Dietary habits were assessed based on the consumption frequency of dairy products, legumes or eggs, meat or fish, and fruits or vegetables, categorized into three levels.

#### 2.2.3. Physical Functioning

Physical functioning was evaluated through three variables: limitations in activities of daily living (ADLs), limitations in instrumental activities of daily living (iADLs), and self-reported limitations in daily activities due to health problems. ADL and iADL scores were calculated based on the number of reported difficulties in specific daily tasks. Self-reported limitations due to health were assessed using a three-category scale.

#### 2.2.4. Physical Health

This category included the number of chronic diseases, self-perceived health, and hospital stays in the last 12 months. The number of chronic diseases was based on self-reported diagnoses and categorized into three levels. Self-perceived health was assessed through a standardized question and classified into three categories. Hospital stays in the past year were recorded as a binary variable.

#### 2.2.5. Mental Health

Mental health variables included quality of life and well-being, depressive symptoms, loneliness, life satisfaction, and social network satisfaction. Quality of life was measured using the CASP-12 scale. Depressive symptoms were evaluated using the EURO-D scale and categorized into presence or absence of depressive symptoms. Loneliness was measured using the Three-Item Loneliness Scale. Life satisfaction and social network satisfaction were assessed through self-reported scores on a 0–10 scale.

#### 2.2.6. Living Conditions

This category included two variables: area of residence and type of housing. The area of residence was categorized into three groups: urban, town, or rural. The type of housing was classified into four categories based on the structure of the dwelling.

For a detailed breakdown of response options for each variable, please refer to the Supplementary Materials.

### 2.3. Statistical Analysis

A descriptive analysis was performed to estimate the proportion of individuals with polypharmacy across 28 countries. To account for differences in age and gender distributions across the 28 countries included in the study, the prevalence of polypharmacy was standardized to the 2013 European standard population. This was performed using the direct standardization method, applying age- and gender-specific weights to the observed prevalence rates along with corresponding 95% confidence intervals (95% CI), which were calculated.

Given the multilevel structure of the data, a non-conditional multilevel logistic regression model was applied with polypharmacy as the dependent variable. Initial univariate multilevel logistic regression models were conducted to examine the association between each covariate and polypharmacy. Significant covariates were then included in a multivariate non-conditional multilevel logistic regression model. Odds ratios (OR) and their 95% CI were reported.

All analyses were conducted using IBM SPSS (version 29; IBM Corp., Armonk, NY, USA), with a significance level set at 0.05.

## 3. Results

### 3.1. Prevalence Study

**Figure 1:**
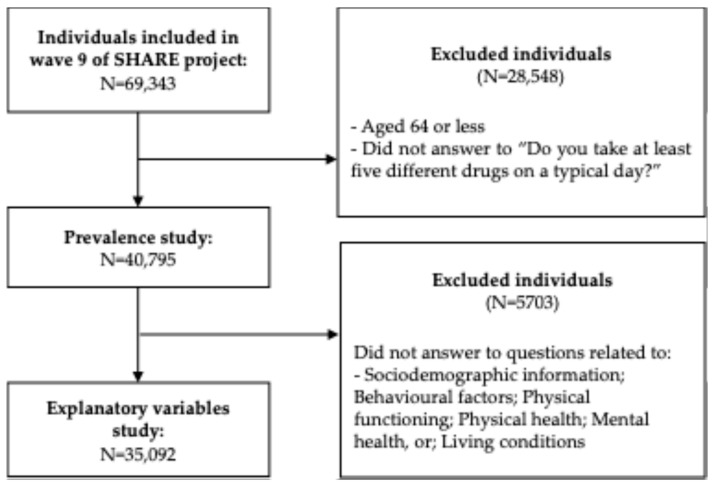
Flow diagram of the individuals’ selection for the prevalence and explanatory variables studies.

**Figure 2:**
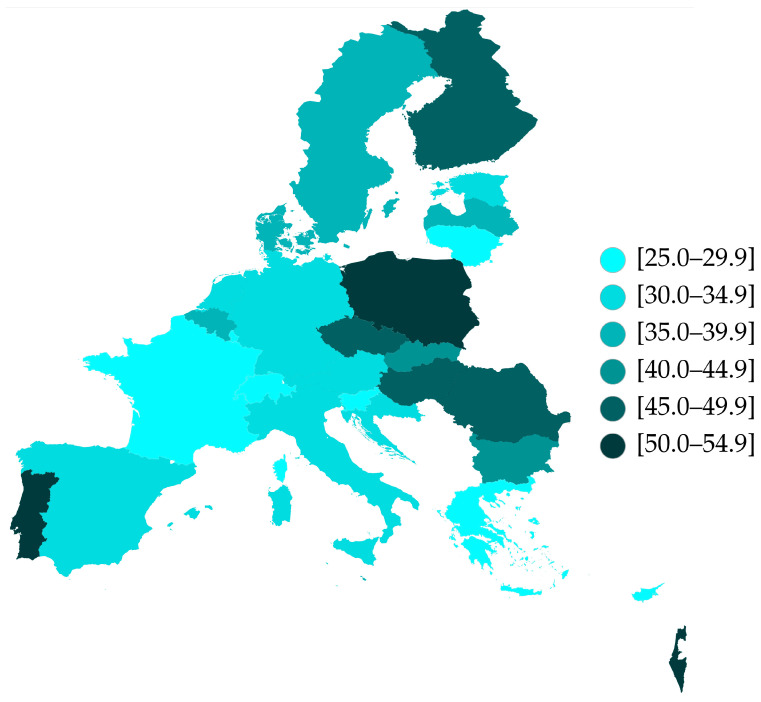
Prevalence of polypharmacy among European older adults (65 years or older) among 27 European countries and Israel.

In this study, we included participants from wave 9 of the SHARE survey who were aged 65 years or older and had no missing values regarding their age, gender, and polypharmacy. This selection resulted in a total of 40,795 participants (Figure 1). We assessed the geographical distribution of polypharmacy across different countries (Table 1), with prevalence rates varying from 25.0% to 51.8%. Slovenia, Greece, and Switzerland had the lowest rates of polypharmacy (25.0%, 25.9%, and 26.0%, respectively), whereas Portugal, Israel, and Poland exhibited the highest prevalence (51.3%, 51.5%, and 51.8%, respectively) (Figure 2). Women from various countries have a polypharmacy prevalence of 36.5%, which is slightly higher than the 35.8% prevalence observed in men. Additionally, there is a direct correlation between age and the prevalence of polypharmacy; as age increases, so does the likelihood of polypharmacy. Among individuals aged 65–74 years, the prevalence is 30.0%. This rises to 41.1% for those aged 75–84 years and reaches 49.4% for individuals aged 85 years and older (Table 1).

### 3.2. Correlations of Polypharmacy with Explanatory Variables

To examine the relationship between polypharmacy and various explanatory variables, we selected participants from the prevalence study who had completed questions regarding their sociodemographic information (marital status, years of education, financial constraints), their usual behavior (smoking habits, alcohol consumption, participation in vigorous and moderate physical activities, as well as the frequency of their intake of dairy products, legumes or eggs, meat, fish or chicken, and fruits or vegetables), their physical functioning (limitations in performing activities of daily living and instrumental activities of daily living, health-related restrictions on activities), physical health (self-perceived health status, the number of chronic conditions, hospital stays over the past 12 months), mental health (quality of life and well-being, depression, loneliness, life satisfaction, network satisfaction), and their living condition (area of building and type of building in which they reside). This process yielded a total of 35,092 participants (Table 2), of whom 20,050 (71.4%) were female. By analyzing data across all participating countries with unadjusted models, we identified a significant association between polypharmacy and most of the exploratory variables included in this study. The variable area of building was the only variable that did not have an association with polypharmacy (Table 2).

In the adjusted model, we found a significant association between 3 variables in the sociodemographic group. Age was statistically significantly associated with polypharmacy; specifically, as people’s ages increased, so did the prevalence of polypharmacy. The odds ratios (OR) were 1.196 (95% CI: 1.132–1.265) for individuals aged 75–84 years and 1.163 (95% CI: 1.066–1.268) for those aged 85 years and older. Shortage of money was identified as a variable, with statistical significance only for individuals who reported experiencing this “sometimes” [OR = 0.927; (0.861–0.998)].

In the group of behavioral factors, five variables were associated with polypharmacy. A decrease in participation in vigorous sports or activities was linked to a higher prevalence of polypharmacy [OR = 1.209 (95% CI: 1.124–1.300)]. Consuming at least one alcoholic beverage in the past week showed a statistically significant inverse association with polypharmacy [OR = 0.790 (95% CI: 0.745–0.837)]. Additionally, engaging in moderate-energy activities was associated with polypharmacy, with odds ratios varying based on frequency: OR = 1.025 (95% CI: 0.949–1.107) for “once a week”, OR = 1.139 (95% CI: 1.032–1.258) for “one to three times a month”, and OR = 1.205 (95% CI: 1.100–1.307) for “hardly ever or never”. The frequency of consuming dairy products and legumes or eggs also emerged as independent factors. A consumption of dairy products 3–6 times a week was associated with polypharmacy [OR = 1.073 (95% CI: 1.005–1.146)], whereas a similar frequency of consuming legumes or eggs was inversely associated with polypharmacy [OR = 0.889 (95% CI: 0.809–0.976)].

All three variables in the physical functioning group were positively associated with polypharmacy: limitations in performing activities of daily living, instrumental activities of daily living, and activity limitations due to health issues.

In the physical health group, three variables were also linked to polypharmacy. Self-perceived health categorized as “Good” [OR = 1.313 (95% CI: 1.190–1.450)] and “Fair or Bad” [OR = 2.371 (95% CI: 2.136–2.631)] was significantly associated with polypharmacy. Having “two or more chronic conditions” showed a strong association with polypharmacy [OR = 4.258 (95% CI: 3.650–4.968)]. Additionally, those who “stayed overnight in a hospital in the last 12 months” were 1.561 times (95% CI: 1.458–1.671) more likely to be under polypharmacy.

Among the mental health variables, higher scores in quality of life and well-being, as well as in life satisfaction, were associated with a lower likelihood of polypharmacy [OR = 0.991 (95% CI: 0.983–0.998)] and [OR = 0.980 (95% CI: 0.963–0.998)], respectively. In contrast, depressive symptoms were linked to a higher probability of polypharmacy [OR = 1.282 (95% CI: 1.208–1.362)]. Additionally, network satisfaction was found to be significantly associated with polypharmacy [OR = 1.034 (95% CI: 1.014–1.054)].

Among the living condition variables, individuals living in a farmhouse were less likely to be under polypharmacy [OR = 0.837 (95% CI: 0.751–0.932)], whereas those residing in a building with three or more floors were more likely to be under polypharmacy [OR = 1.088 (95% CI: 1.027–1.153)], compared to those living in “A free-standing one/two-family house OR a one- or two-family house as a row or double house”.

## 4. Discussion

This study aimed to evaluate the prevalence of polypharmacy and the variables associated with it among older adults across 27 European countries and Israel, as included in SHARE Wave 9. The ninth wave of SHARE data collection took place between October 2021 and September 2022, utilizing a Computer-Assisted Personal Interview (CAPI) in the same 28 countries that participated in the eighth wave of the SHARE panel. Wave 9 gathered data from 69,154 individual interviews conducted in 47,957 households, with sample sizes varying by country—from a minimum of 731 observations in Cyprus to a maximum of 4802 observations in Poland. Our study revealed that Slovenia, Greece, and Switzerland demonstrated the lowest rates of polypharmacy, with figures of 25.0%, 25.9%, and 26.0%, respectively. Conversely, Portugal, Israel, and Poland exhibited the highest prevalence of polypharmacy, with rates of 51.3%, 51.5%, and 51.8%, respectively.

In Table 3, we compared the prevalence of polypharmacy across 17 countries included in SHARE Wave 6 with the findings from this study [18]. The sixth wave of SHARE data collection concluded in November 2015. Our results reveal that 11 out of the 17 countries experienced an increase in polypharmacy prevalence between Wave 6 and Wave 9. The countries with the largest increases were Poland, Israel, Portugal, and Denmark. Specifically, Poland’s prevalence rose from 33.8% to 51.8% (an 18% increase), Israel from 37.5% to 51.5% (14% increase), Portugal from 36.9% to 51.3% (14.4% increase), and Denmark from 32.7% to 39.6% (7% increase). Additional countries with rising polypharmacy rates include Germany (1.8% increase, 30.3% to 32.1%), Spain (2.2% increase, 31.6% to 33.8%), Belgium (4.2% increase, 34.0% to 38.2%), Estonia (5.1% increase, 28.4% to 33.5%), Croatia (5.4% increase, 27.3% to 32.7%), the Czech Republic (6.7% increase, 39.9% to 46.6%), and Sweden (6.8% increase, 31.0% to 37.8%). These findings align with studies conducted in the respective countries [19]. For example, research in Israel suggests that the high prevalence of polypharmacy reflects increased life expectancy and rapid population growth [20]. Additionally, polypharmacy growth across Europe may be attributed to the lack of national-scale medication adherence assessments in most European countries [21]. It is important to consider the timeline between SHARE Waves 6 and 9, which includes the COVID-19 pandemic. Evidence indicates that older adults were more likely to develop symptomatic infections, with comorbid patients being at higher risk of severe illness and mortality [22,23]. A study across Europe and Israel found that healthcare access deteriorated during the pandemic, particularly for older adults. This unmet healthcare demand may have increased health vulnerabilities, exacerbating chronic conditions and leading to significant negative health outcomes and increased mortality [24,25,26]. Conversely, several countries observed a decrease in polypharmacy prevalence, including Italy (0.2% decrease, 32.9% to 32.7%), Austria (0.3% decrease, 31.5% to 31.2%), Switzerland (0.3% decrease, 26.3% to 26.0%), Luxembourg (0.3% decrease, 29.5% to 29.2%), France (2.3% decrease, 31.0% to 28.7%), Slovenia (3.1% decrease, 28.1% to 25.0%), and Greece (3.4% decrease, 29.3% to 25.9%). In France, this decline has been linked to targeted measures aimed at reducing polypharmacy in older adults, such as ongoing physician training, electronic clinical decision support systems, and pharmacist interventions [27,28]. These strategies have proven to be effective tools in mitigating polypharmacy exposure [29].

Research has consistently shown that polypharmacy, defined as the concurrent use of multiple medications, is highly prevalent among older adults. Prevalence rates range from 27% to 59% in primary care settings and between 46% and 84% in hospitalized patients [30]. A study across 18 European countries identified a prevalence range of 26.3% to 39.9% [31]. In the United States, a nationwide study noted an increase in polypharmacy among older adults, with rates rising from 30.6% to 35.8% [32]. Another investigation revealed that approximately 37% of the general population experiences polypharmacy. This condition is particularly pronounced in certain subgroups: around 45% of individuals aged over 65 are affected, compared to younger adults, with prevalence reaching 52% among hospitalized patients [33].

In our study, we observed similar patterns regarding the prevalence of polypharmacy among the older population. For those aged 65–74 years, the prevalence globally was 30.0%; for those aged 75–84 years, it was 41.1%; and for those aged 85 and older, it was 49.9%. Polypharmacy was more prevalent in women than in men, affecting 71.4% of females compared to 42.9% of males. A study in Israel reported similar findings, with prevalence rates of 66.4% for women and 61.6% for men regarding polypharmacy [34]. Likewise, the overall prevalence of polypharmacy among adults aged 65 and older in Spain was 23.2%, higher in women (28.1%) than in men (17.2%) [35]. This may relate to the fact that women and men respond to drug therapies differently due to sex and gender differences [36]. Gender-related sociocultural factors include the fact that women consult physicians more frequently and are generally more concerned about their health. Older women are more likely to be caregivers and to have responsibility for supervising the medications for a spouse or an older family member [37]. When older women themselves require assistance with their medications, they might be less likely to have a spouse or a partner to help provide that supervision [38]. Biological sex differences can also partly explain these polypharmacy prevalence differences. For instance, women may face a greater vulnerability compared to men due to their higher subcutaneous fat and surface-to-mass ratio [39,40]. Thus, they tend to seek health services more often and earlier than men, and they are also more accustomed to taking medications [41,42,43]. Additionally, women recognize and experience more health problems along with greater underlying gender-related psycho-social and behavioral influences, leading them to perceive more symptoms than men [44,45].

In this study, we found no association between marital status and years of education with polypharmacy. This suggests that the relationship between polypharmacy and low education levels may be explained by a higher prevalence of diseases among individuals with lower socioeconomic status compared to the general population [46]. Furthermore, our findings indicated that individuals who occasionally experienced financial difficulties appeared to be more susceptible to polypharmacy. In other studies, it was found that those who reported a lack of financial resources seemed to be at greater risk for this condition [47].

This study found that alcohol consumption is statistically significantly associated with polypharmacy. Literature shows that 93% of alcohol drinkers take at least one chronic medication that may interact with alcohol. Additionally, 42% of these individuals use at least one chronic drug for which alcohol consumption is considered contraindicated [48]. Both alcohol use and medication use are on the rise, and both can lead to hepatotoxicity while contributing to various conditions that result in disability. Alcohol consumption can also increase the risk of falls and enhance the effects of several medications [48,49]. Also, this can be caused by the ‘healthy drinker bias’, wherein moderate alcohol consumption is linked to healthier lifestyles and better overall health, reducing the need for multiple medications. However, this finding is not generalizable and must be interpreted cautiously. Interestingly, the study found that individuals who smoke daily are less likely to experience polypharmacy. A study carried out in 2018 found no statistically significant association between smoking and polypharmacy [50]. Similarly, another study found that smoking was not associated with excessive polypharmacy in older polypharmacy patients [51]. However, other research shows that there is a positive association between smoking and polypharmacy [52,53]. The reason for this is that smoking contributes to the burden of disease. Smoking is linked to an increase in cardiovascular diseases, respiratory diseases, and also the risk of mortality [54].

It is widely recognized that physical activity levels tend to decline with age, which, in turn, increases the prevalence of polypharmacy. Gharekhani et al. discovered that middle-aged and older adults with moderate to low physical activity levels had 47% and 90% higher odds of experiencing polypharmacy, respectively [55]. Similarly, Bueno et al. reported that lower levels of locomotion and leisure-time physical activity were linked to higher chances of polypharmacy among middle-aged and older adults with hypertension [56]. There exists a bidirectional relationship between polypharmacy and physical function in older adults [57]. It is essential to note that polypharmacy is associated with several negative health-related outcomes, such as falls [58], disability [59], and poorer physical function [57], all of which can lead to a reduction in physical activity among older adults. Furthermore, older individuals using multiple medications are more likely to experience side effects, particularly those affecting physical health, which can reduce their physical activity and increase sedentary behavior. For instance, statins can cause severe side effects such as myalgia and rhabdomyolysis, beta blockers alter local muscular metabolic properties and hinder endurance exercise, and various drugs, including corticosteroids and chemotherapy medications, are associated with muscle weakness and wasting [60,61,62,63,64].

The presence of multiple concurrent diseases in older adults complicates the development of an optimized medication regimen. It is crucial to balance the number of medications and their potential benefits against their adverse effects. Consequently, as the number of medications increases, the likelihood of polypharmacy and functional impairment also rises [57]. A key determinant of physical function is an individual’s clinical condition, which includes cognitive and neuromuscular function, orthopedic issues, and symptoms such as fatigue, pain, and shortness of breath [65]. Impairments in physical function often result from a deterioration in clinical status, typically following targeted medication treatments aimed at restoring health. This highlights why an individual’s concurrent diseases or clinical status are primary determinants of the medications required and contribute significantly to the issue of polypharmacy [66]. In fact, this study found a positive correlation between limited activities because of health conditions and polypharmacy.

Research indicates that polypharmacy and potentially inappropriate pharmacotherapy can lead to disabilities in activities of daily living (ADLs) among older individuals [67]. In this study, we identified a direct association between difficulties in daily living and polypharmacy. Other research suggests that the relationship between polypharmacy and decline in functional status may operate through several pathways. First, most individuals taking more than six medications often suffer from multiple chronic diseases, which are strong predictors of health outcomes. Second, polypharmacy can frequently result in kidney and liver toxicity. Lastly, certain medications can impact cognitive functions and balance centers in the brain, increasing the risk of falls and subsequent fractures [68]. Consistent with this, studies have shown that patients using five or more medications are at a higher risk of frailty, functional decline, and falls [69,70]. In addition to ADLs, it is recognized that IADL disability is common among older adults living in the community. This study also found a positive association between IADL disability and polypharmacy. One study indicated that the point prevalence estimates of IADL disability were 34% at baseline and 37.4% at follow-up, which falls within the broader range of 13.9% to 53.5% reported in other studies [71,72,73,74,75].

Other studies show that excessive polypharmacy is associated with a decline in nutritional status, functional ability, and cognitive capacity in older persons aged 75 years and older [76,77]. The role of polypharmacy in nutritional status appears crucial; a clear association between polypharmacy and malnutrition has been widely reported in older individuals [78]. Nutritional status results from the balance between caloric intake and energy expenditure incurred by the body to ensure vital processes. Hence, malnutrition is a condition characterized by an imbalance in energy, protein, or other nutrients responsible for adverse effects on body composition, physical function, and clinical outcomes [79]. In this study, nutrition was assessed by checking how often individuals consumed dairy products and legumes or eggs. These variables were statistically significantly associated with polypharmacy when individuals answered that they consumed these products 3–6 times a week. This shows that a healthy lifestyle, which consists of eating a balanced diet every day, leads to a decrease in polypharmacy prevalence. In this study, nutrition was also assessed by checking how often individuals consume meat, fish, chicken, fruits, or vegetables. However, these variables did not result in a positive association with polypharmacy.

This study found that people with poor self-perceived health had a higher risk of having polypharmacy. In fact, non-polypharmacy users perceived their health status to be better than polypharmacy users [80]. Hospital admission represents a notable disadvantage of polypharmacy. This study revealed a positive correlation between the variable ‘hospital stay for at least one night in the past 12 months and polypharmacy. Existing literature has established that polypharmacy is associated with a significantly heightened risk of hospitalization and mortality. Consequently, polypharmacy has detrimental effects on the health and survival of older individuals [81]. Both multimorbidity and polypharmacy were identified as factors related to admissions resulting from adverse drug reactions (ADRs) in univariate analyses. On average, patients experiencing an ADR were prescribed 35% more medications than those who did not [82]. Furthermore, research conducted by Perreault et al. indicated that polypharmacy is linked to a 31% increased risk of mortality. Their study, which concentrated on patients with heart failure, highlighted that polypharmacy is highly prevalent among older adults hospitalized for this condition, with prevalence rates ranging from 84% to 95% between 2003 and 2014 [83,84].

According to the World Health Organization, quality of life (QoL) is defined as an individual’s perception of their position in life, considering the cultural and value systems in which they exist, as well as their goals, expectations, standards, and concerns [85]. QoL is a vital component of successful aging, as low quality of life during this phase is linked to reduced activity and physical capacity, an increase in chronic diseases, and social isolation [86,87]. An increasing number of symptoms can significantly contribute to the decline of QoL [88]. Polypharmacy can negatively impact a patient’s well-being due to the complications of managing multiple medications, their side effects, and the stigma associated with their use [68]. One tool used to assess the quality of life in older adults is the CASP scale, which evaluates four domains: Control, Autonomy, Self-Realization, and Pleasure. The standard scale comprises 19 items, but a shortened version with just 12 items is utilized in the SHARE questionnaire [89]. This study indicates that quality of life and well-being can serve as predictors of polypharmacy; lower levels of quality of life and well-being are associated with an increased use of medications. Additionally, family networks and friendships provide essential support for older adults. Numerous studies have highlighted the significant benefits of these relationships [90,91,92]. Our findings also reveal a positive correlation between lower satisfaction with social networks and life satisfaction with elevated medication use.

We discovered a connection between depression and polypharmacy. Depression negatively affects overall health, making individuals with depression more susceptible to polypharmacy. This link has been well-established by previous researchers [93,94]. Additionally, depression can also result from polypharmacy, rather than just being a predictor [95]. We did not find any association between polypharmacy and loneliness. A study carried out in Turkey found that loneliness and social isolation were higher in older adults with polypharmacy than in those without polypharmacy [96]. Similarly, a study carried out in Sweden found that polypharmacy was associated with the occurrence of loneliness and social isolation among older adults [97]. Another study conducted in 2013 reported that older adults with polypharmacy develop negative emotions and experience emotional loneliness because there is no one around to help minimize polypharmacy [98].

In this study, we found a connection between the type of building individuals live in and the prevalence of polypharmacy. Notably, we discovered a positive correlation among individuals who reported living in a farmhouse or in a building with three or more floors. This trend may be attributed to these residents being exposed to higher levels of pollution, which could increase their risk of allergies. Conversely, we found no association between individuals residing in housing complexes designed for the older population or in nursing homes and polypharmacy. This finding contrasts with existing literature, which typically indicates a high rate of polypharmacy among nursing home residents [47,98,99,100,101].

This study has several limitations that should be considered. First, the potential influence of confounding factors, such as socioeconomic status, healthcare access, and comorbidities, cannot be entirely ruled out despite adjustments for key demographic and health-related variables. Additionally, the reliance on self-reported data introduces biases, including recall and social desirability bias, which may lead to inaccuracies in medication reporting. The SHARE data, being self-reported, could also be subject to selection bias, as individuals who participate in such surveys are often healthier and more motivated, potentially excluding a substantial proportion of older adults with significant comorbidities or cognitive impairments. Future studies utilizing objective prescription or pharmacy records could address these limitations.

The study also does not account for the role of caregivers, who are critical in managing the healthcare of older adults. The use of cross-sectional data further limits the ability to establish causal relationships or understand temporal patterns. Moreover, the absence of qualitative insights into medication adherence represents a notable gap, as this study could not explore the lived experiences, challenges, or perceptions of older individuals regarding their medication use. Integrating qualitative approaches, such as interviews or focus groups, in future research could provide a more comprehensive understanding of adherence behaviors and barriers. Finally, the findings are based on a sample from European countries and Israel, which may limit their generalizability to other populations. Future studies should consider broader geographic and cultural contexts to enhance the applicability of results.

Several strengths of the study should be emphasized. First, it involved a large number of participants and had an international cross-sectional design. This approach enables comparisons and offers insights for stakeholders from a broader perspective. Additionally, this study is among the first to examine self-care management regarding over-the-counter (OTC) drugs and dietary supplements, including vitamins and minerals. This focus sheds light on a growing trend in industrialized countries that warrants attention from healthcare providers. Recently, there has been an increase in the use of dietary supplements in several European countries [102].

## 5. Conclusions

In conclusion, our results indicate that polypharmacy is a widespread issue among older adults, with prevalence rates ranging from 25.0% to 51.8% in Europe and Israel. Furthermore, polypharmacy is a multifactorial condition, associated with a variety of sociodemographic and behavioral factors, as well as aspects of physical functioning, physical health, mental health, and living conditions. Identifying the variables related to polypharmacy is crucial for recognizing and monitoring older groups that are most vulnerable to this issue. Interventions designed to reduce polypharmacy should consider the diverse factors linked to this condition.

## Figures and Tables

**Table 1:** Prevalence of polypharmacy detailed by country, age, and gender among the 28 countries included in SHARE’s wave 9.

	Overall (95% CI)	Overall			Male (95% CI)	Male			Female (95% CI)	Female		
		Standardized Rates (95% CI)				Standardized Rates (95% CI)				Standardized Rates (95% CI)		
		65–74 yrs	75–84 yrs	≥85 yrs		65–74 yrs	75–84 yrs	≥85 yrs		65–74 yrs	75–84 yrs	≥85 yrs
Austria	31.2 [30.7–31.8]	22.2 [21.5–22.8]	39.6 [38.5–40.7]	47.5 [45.6–49.4]	31.4 [30.7–32.2]	24.1 [23.1–25.0]	38.0 [36.5–39.5]	45.3 [42.7–48.0]	31.0 [30.2–31.8]	20.8 [19.9–21.7]	40.6 [39.0–42.2]	48.8 [46.1–51.6]
Belgium	38.2 [37.6–38.8]	33.8 [33.0–34.6]	41.4 [40.3–42.6]	48.5 [46.6–50.4]	36.1 [35.3–37.0]	32.3 [31.2–33.4]	38.5 [37.0–40.0]	46.2 [43.5–48.9]	39.9 [39.0–40.8]	35.1 [34.0–36.3]	43.7 [42.1–45.3]	49.8 [47.1–52.6]
Bulgaria	42.9 [42.2–43.5]	38.0 [37.5–39.2]	48.1 [46.9–49.3]	48.1 [46.2–50.1]	41.0 [40.1–41.9]	36.6 [35.4–37.7]	42.9 [41.3–44.5]	54.5 [51.7–57.5]	43.9 [43.0–44.9]	39.4 [38.2–40.6]	51.3 [49.6–53.1]	43.8 [41.2–46.4]
Croatia	32.7 [32.1–33.2]	26.0 [25.3–26.7]	40.0 [39.0–41.2]	41.4 [39.6–43.2]	30.2 [29.5–31.0]	24.3 [23.4–25.2]	36.6 [35.1–38.1]	38.6 [36.2–41.2]	34.5 [33.7–35.3]	27.5 [26.5–28.5]	42.6 [41.0–44.2]	43.0 [40.4–45.6]
Cyprus	27.1 [26.6–27.7]	19.7 [19.1–20.3]	31.4 [30.5–32.4]	47.1 [45.3–49.1]	28.3 [27.5–29.0]	23.3 [22.4–24.3]	29.6 [28.3–30.9]	45.5 [42.9–48.2]	26.3 [25.6–27.0]	16.9 [16.2–17.8]	32.8 [31.4–34.2]	48.6 [46.0–51.5]
CzechRepublic	46.6 [45.9–47.3]	41.1 [40.2–42.0]	50.1 [48.9–51.3]	60.6 [58.4–62.7]	47.3 [46.3–48.3]	41.2 [40.0–42.5]	50.7 [49.0–52.4]	64.0 [60.9–67.2]	46.2 [45.3–47.2]	41.0 [39.8–42.3]	49.8 [48.1–51.5]	58.7 [55.8–61.8]
Denmark	39.6 [39.0–40.2]	37.0 [36.2–37.9]	41.0 [39.9–42.1]	46.5 [44.6–48.4]	41.1 [40.2–42.0]	36.7 [35.5–37.8]	42.3 [40.8–43.9]	56.4 [53.5–59.4]	38.5 [37.6–39.4]	37.4 [36.2–38.5]	39.7 [38.2–41.3]	40.2 [37.8–42.8]
Estonia	33.5 [32.9–34.0]	27.9 [27.2–28.6]	39.2 [38.2–40.3]	41.7 [39.9–43.5]	32.8 [32.0–33.6]	27.2 [26.2–28.2]	36.5 [35.1–38.0]	46.3 [43.7–49.0]	33.9 [33.1–34.7]	28.4 [27.3–29.4]	40.5 [39.0–42.1]	39.9 [37.5–42.5]
Finland	45.5 [44.9–46.2]	36.4 [35.6–37.2]	52.5 [51.3–53.8]	65.8 [63.6–68.1]	45.4 [44.5–46.4]	36.0 [ 34.9–37.2]	55.1 [53.3–57.0]	59.6 [56.6–62.7]	45.5 [44.6–46.5]	36.8 [35.6–37.9]	50.2 [48.5–52.0]	70.0 [66.8–73.4]
France	28.7 [28.1–29.2]	21.1 [20.5–21.8]	35.8 [34.8–36.9]	41.7 [39.9–43.5]	31.2 [30.4–32.0]	26.1 [25.1–27.1]	34.8 [33.3–36.2]	43.2 [40.6–45.8]	26.7 [26.0–27.4]	17.2 [16.4–18.0]	36.6 [35.1–38.1]	40.9 [38.4–43.5]
Germany	32.1 [31.5–32.7]	23.6 [22.9–24.2]	38.5 [37.5–39.6]	51.1 [49.1–53.1]	34.2 [33.4–35.1]	26.0 [25.1–27.0]	41.8 [40.3–43.4]	48.9 [46.2–51.7]	30.1 [29.3–30.9]	21.4 [20.5–22.3]	35.3 [33.9–36.8]	53.2 [50.4–56.2]
Greece	25.9 [25.4–26.4]	16.3 [15.8–16.9]	30.1 [29.1–31.0]	55.2 [53.1–57.3]	22.9 [22.2–23.6]	15.1 [14.4–15.9]	25.3 [24.1–26.6]	49.2 [46.5–52.0]	28.3 [27.6–29.1]	17.2 [16.4–18.0]	34.3 [32.9–35.7]	59.3 [56.3–62.4]
Hungary	47.6 [46.9–48.3]	41.4 [40.5–42.2]	58.2 [56.9–59.5]	46.0 [44.2–48.0]	46.1 [45.1–47.0]	39.2 [38.0–40.4]	58.6 [56.8–60.5]	42.1 [39.6–44.7]	48.5 [47.6–49.5]	43.0 [41.7–44.2]	57.8 [56.0–59.7]	47.7 [45.1–50.5]
Israel	51.5 [50.8–52.2]	44.7 [43.8–45.6]	55.9 [54.6–57.2]	68.4 [66.1–70.7]	51.4 [50.4–52.4]	46.6 [45.3–47.9]	54.9 [53.2–56.8]	62.1 [59.0–65.2]	51.9 [50.9–52.9]	43.5 [42.2–44.8]	56.7 [54.9–58.5]	74.6 [71.2–78.0]
Italy	32.7 [32.1–33.3]	24.8 [24.2–25.5]	38.7 [37.6–39.8]	50.0 [48.1–52.0]	34.5 [33.7–35.3]	25.8 [24.8–26.7]	42.3 [40.7–43.9]	51.0 [48.2–53.9]	31.2 [30.4–32.0]	24.1 [23.2–25.1]	35.7 [34.3–37.2]	49.3 [46.6–52.1]
Latvia	38.5 [37.9–39.2]	30.8 [30.1–31.6]	48.6 [47.4–49.8]	44.8 [43.0–46.7]	31.5 [30.7–32.3]	25.7 [24.8–26.7]	39.0 [37.5–40.5]	36.4 [34.0–38.8]	42.0 [41.1–42.9]	33.8 [32.7–34.9]	52.9 [51.1–54.7]	48.2 [45.5–51.0]
Lithuania	27.2 [26.7–27.7]	21.2 [20.6–21.9]	33.0 [32.0–34.0]	37.0 [35.3–38.7]	25.5 [24.8–26.3]	23.7 [22.7–24.6]	26.7 [25.5–28.0]	30.3 [28.2–32.5]	27.8 [27.0–28.5]	19.9 [19.1–20.8]	36.1 [34.7–37.6]	39.0 [36.6–41.6]
Luxembourg	29.2 [28.6–29.7]	25.5 [24.8–26.2]	35.8 [34.8–36.9]	27.3 [25.8–28.8]	34.0 [33.1–34.8]	33.6 [32.5–34.7]	34.8 [33.4–36.2]	33.3 [31.1–35.7]	25.1 [24.4–25.8]	18.9 [18.0–19.7]	37.0 [35.6–38.5]	20.0 [18.3–21.8]
Malta	36.3 [35.7–36.9]	29.0 [28.3–29.7]	43.6 [42.5–44.8]	48.1 [46.2–50.1]	38.9 [38.0–39.8]	33.3 [32.2–34.5]	43.6 [42.0–45.2]	50.0 [47.3–52.9]	34.0 [33.2–34.9]	25.0 [24.1–26.0]	43.6 [42.0–45.3]	47.1 [44.4–49.8]
The Netherlands	32.0 [31.5–32.6]	29.0 [28.-29.7]	34.0 [33.0–35.0]	39.7 [38.0–41.5]	33.4 [32.6–34.2]	30.4 [29.4–31.5]	36.4 [34.9–37.9]	38.1 [35.7–40.6]	30.8 [30.0–31.6]	27.8 [26.8–28.8]	31.7 [30.4–33.1]	41.1 [38.6–43.7]
Poland	51.8 [51.1–52.5]	43.6 [42.7–44.5]	57.7 [56.4–59.0]	70.5 [68.2–72.9]	49.9 [48.9–50.9]	43.5 [42.3–44.8]	51.9 [50.2–53.7]	71.7 [68.4–75.1]	53.1 [52.0–54.1]	43.7 [42.5–45.0]	61.8 [59.9–63.7]	69.7 [66.5–73.1]
Portugal	51.3 [50.6–52.0]	45.4 [44.5–46.3]	55.1 [53.8–56.4]	66.2 [64.0–68.5]	45.9 [44.9–46.8]	41.0 [39.8–42.3]	43.4 [41.8–45.0]	72.7 [69.4–76.1]	55.7 [54.6–56.7]	49.1 [47.8–50.5]	64.1 [62.1–66.1]	61.4 [58.3–64.5]
Romania	48.0 [47.3–48.7]	45.5 [ 44.6–46.4 ]	50.8 [49.6–52.0]	51.3 [49.3–53.3]	44.6 [43.7–45.5]	38.2 [37.0–39.4]	51.5 [49.7–53.2]	53.6 [50.7–56.5]	50.3 [49.3–51.3]	50.3 [49.0–51.7]	50.3 [48.6–52.1]	50.0 [47.3–52.9]
Slovakia	41.0 [40.3–41.6]	32.0 [31.2–32.7]	44.3 [43.2–45.5]	70.0 [67.7–72.4]	35.7 [34.9–36.6]	31.5 [ 30.5–32.6]	37.0 [35.5–38.5]	50.0 [47.3–52.9]	43.9 [43.0–44.9]	32.4 [31.3–33.5]	49.3 [47.6–51.0]	78.6 [75.1–82.1]
Slovenia	25.0 [24.5–25.5]	18.5 [17.9–19.1]	28.4 [27.5–29.3]	43.7 [41.9–45.6]	25.7 [25.0–26.4]	19.0 [18.2–19.9]	29.2 [27.9–30.6]	44.3 [41.7–47.0]	24.5 [23.8–25.2]	18.0 [17.2–18.8]	27.7 [26.5–29.0]	43.4 [40.9–46.1]
Spain	33.8 [33.3–34.4]	24.9 [24.2–25.6]	41.6 [40.5–42.7]	51.3 [49.3–53.3]	31.4 [30.6–32.2]	23.9 [22.9–24.8]	38.9 [37.4–40.5]	43.4 [40.9–46.1]	35.7 [34.8–36.5]	25.8 [24.8–26.7]	43.5 [41.9–45.2]	56.8 [53.8–59.8]
Sweden	37.8 [37.2–38.4]	34.2 [ 33.4–35.0 ]	40.3 [39.2–41.4]	46.6 [44.7–48.5]	38.8 [37.9–39.7]	33.0 [31.9–34.1]	45.8 [44.1–47.4]	45.2 [42.6–47.9]	36.8 [36.0–37.7]	35.1 [34.0–36.2]	35.4 [34.0–36.9]	47.7 [45.1–50.5]
Switzerland	26.0 [25.5–26.5]	22.3 [21.6–22.9]	26.4 [25.5–27.3]	40.7 [ 38.9–42.5]	28.3 [27.6–29.1]	24.3 [23.4–25.3]	30.8 [29.5–32.2]	38.8 [36.4–41.3]	23.9 [23.2–24.6]	20.6 [19.7–21.5]	22.4 [21.3–23.6]	41.8 [39.3–44.4]
Total	36.2 [35.6–36.8]	30.0 [29.3–30.8]	41.1 [40.0–42.2]	49.4 [47.4–51.4]	35.8 [34.9–36.6]	29.9 [28.9–31.0]	40.1 [38.6–41.7]	48.8 [46.1–51.6]	36.5 [35.7–37.4]	30.1 [29.1–31.2]	41.8 [40.3–43.4]	49.7 [47.0–52.6]

**Table 2:** Association of explanatory variables with polypharmacy; unadjusted and adjusted models.

		*N*	*N* (%) Polypharmacy	Unadjusted Model			Adjusted Model		
		35,092	12,459 (35.5)	OR	CI 95	*p*	OR	CI 95	*p*
Sociodemographics	Gender								
	Male	15,042	5213 (34.7)	1	-	-	1	-	-
	Female	20,050	7246 (36.1)	1.057	1.011–1.106	0.016	0.808	0.766–0.854	<0.001
	Age								
	65–74 years	18,042	5343 (29.6)	1	-	-	1	-	-
	75–84 years	13,050	5213 (39.9)	1.650	1.572–1.733	<0.001	1.196	1.132–1.265	<0.001
	≥85 years	4000	1903 (47.6)	2.369	2.206–2.545	<0.001	1.163	1.066–1.268	<0.001
	Marital status								
	Never married	1360	459 (33.8)	1	-	-			
	Married or registered partnership	23,061	7609 (33.0)	0.929	0.827–1.045	0.220			
	Divorced	2623	944 (36.0)	1.041	0.906–1.197	0.570			
	Widowed	8048	3447 (42.8)	1.397	1.236–1.579	<0.001			
	Years of education								
	Low education (0–8)	10,131	3992 (39.4)	1	-	-			
	Moderate education (9–12)	13,501	4839 (35.8)	0.768	0.725–0.814	<0.001			
	High education (13–16)	8234	2659 (32.3)	0.640	0.599–0.684	<0.001			
	Very high education (17+)	3226	969 (30.0)	0.575	0.526–0.629	<0.001			
	Shortage of money								
	Never	13,314	4526 (34.0)	1	-	-	1	-	-
	Rarely	7897	2699 (34.2)	0.969	0.912–1.029	0.299	0.962	0.897–1.032	0.279
	Sometimes	8575	3005 (35.0)	1.054	0.992–1.121	0.088	0.927	0.861–0.998	0.043
	Often	5306	2229 (42.0)	1.553	1.445–1.670	<0.001	1.007	0.920–1.103	0.878
Behavioral Factors	Ever smoked daily								
	No	21,685	7650 (35.3)	1	-	-			
	Yes	13,407	4809 (35.9)	0.692	0.6600.725	<0.001			
	At least one alcoholic beverage in the last 7 days								
	No	19,497	7943 (40.7)	1	-	-	1	-	-
	Yes	15,595	4516 (29.0)	0.584	0.556–0.613	<0.001	0.790	0.745–0.837	<0.001
	Sports or activities that are vigorous								
	More than once a week	8533	2093 (24.5)	1	-	-	1	-	-
	Once a week	4592	1172 (25.5)	1.074	0.987–1.168	0.096	0.918	0.836–1.008	0.072
	One to three times a month	3823	1020 (26.7)	1.166	1.066–1.275	<0.001	0.960	0.868–1.061	0.419
	Hardly ever or never	18,144	8174 (45.1)	2.576	2.427–2.733	<0.001	1.209	1.124–1.300	<0.001
	Activities requiring a moderate level of energy								
	More than once a week	21,645	6305 (29.1)	1	-	-	1	-	-
	Once a week	4891	1708 (34.9)	1.336	1.249–1.429	<0.001	1.025	0.949–1.107	<0.001
	One to three times a month	2614	1073 (41.0)	1.757	1.612–1.914	<0.001	1.139	1.032–1.258	<0.001
	Hardly ever or never	5942	3373 (56.8)	3.352	3.151–3.567	<0.001	1.205	1.110–1.307	<0.001
	How often consume a serving of dairy products								
	Every day	20,791	7153 (34.4)	1	-	-	1	-	-
	3–6 times a week	8352	3062 (36.7)	1.075	1.016–1.138	0.013	1.073	1.005–1.146	0.035
	Less than 3 times a week	5949	2244 (37.7)	1.129	1.060–1.202	<0.001	1.069	0.994–1.150	0.074
	How often consume a serving of legumes or eggs								
	Every day	3561	1299 (36.5)	1	-	-	1	-	-
	3–6 times a week	11,864	4007 (33.8)	0.955	0.880–1.036	0.270	0.889	0.809–0.976	0.014
	Less than 3 times a week	19,667	7153 (36.4)	1.110	1.025–1.201	0.010	0.977	0.892–1.070	0.620
	How often consume a serving of meat, fish or chicken								
	Every day	10,611	3771 (35.5)	1	-	-			
	3–6 times a week	17,602	6177 (35.1)	0.999	0.945–1.057	0.982			
	Less than 3 times a week	6879	2511 (36.5)	1.123	1.048–1.205	0.001			
	How often consume a serving of fruits or vegetables								
	Every day	26,351	9000 (34.2)	1	-	-			
	3–6 times a week	6588	2551 (38.7)	1.118	1.054–1.186	<0.001			
	Less than 3 times a week	2153	908 (42.2)	1.309	1.194–1.435	<0.001			
Physical functioning	Number of limitations performing activities of daily living (ADLs)								
	0	30,573	9563 (31.3)	1	-	-	1	-	-
	1	2352	1335 (56.8)	2.932	2.689–3.197	<0.001	1.209	1.094–1.336	<0.001
	2 or more	2167	1561 (72.0)	5.798	5.255–6.398	<0.001	1.425	1.260–1.611	<0.001
	Number of limitations performing instrumental activities of daily living (iADLs)								
	0	26,954	7601 (28.2)	1	-	-	1	-	-
	1	3246	1609 (49.6)	2.575	2.388–2.775	<0.001	1.351	1.241–1.472	<0.001
	2 or more	4892	3249 (66.4)	5.346	5.002–5.715	<0.001	1.638	1.494–1.797	<0.001
	Limited in activities because of health								
	Not limited	15,005	2868 (19.1)	1	-	-	1	-	-
	Limited, but not severely	13,428	5473 (40.8)	2.959	2.802–3.125	<0.001	1.503	1.411–1.601	<0.001
	Severely limited	6659	4118 (61.8)	7.483	7.001–7.998	<0.001	2.371	2.136–2.631	<0.001
Physical health	Self-perceived health								
	Excellent or very good	4768	693 (14.5)	1	-	-	1	-	-
	Good	13,762	3264 (23.7)	1.957	1.785–2.146	<0.001	1.313	1.190–1.450	<0.001
	Fair or Poor	16,562	8502 (51.3)	7.479	6.832–8.187	<0.001	2.371	2.136–2.631	<0.001
	Number of chronic conditions								
	0	2167	203 (9.4)	1	-	-	1	-	-
	1	8620	1133 (13.1)	0.288	0.245–0.337	<0.001	1.043	0.885–1.229	0.614
	2 or more	24,305	11,123 (45.8)	1.646	1.420–1.908	<0.001	4.258	3.650–4.968	<0.001
	Stayed overnight in the hospital in the last 12 months								
	No	29,511	9451 (32.0)	1	-	-	1	-	-
	Yes	5581	3008 (53.9)	2.545	2.398–2.701	<0.001	1.561	1.458–1.671	<0.001
Mental health	Quality of life and well-being								
	CASP	35,092	12,459 (35.5)	1.046	1.040–1.052	<0.001	0.991	0.983–0.998	0.011
	Depression scale EURO-D								
	Without depressive symptoms	24,279	9451 (38.9)	1	-	-	1	-	-
	With depressive symptoms	10,813	3008 (27.8)	2.589	2.468–2.716	<0.001	1.282	1.208–1.362	<0.001
	Loneliness								
	Without signs of loneliness	28,712	9416 (32.8)	1	-	-			
	With signs of loneliness	6380	3043 (47.7)	1.922	1.816–2.034	<0.001			
	Life satisfaction								
	Life satisfaction	35,092	12,459 (35.5)	0.818	0.807–0.829	<0.001	0.980	0.963–0.998	0.028
	Network satisfaction								
	Network satisfaction	35,092	12,459 (35.5)	0.960	0.944–0.976	<0.001	1.034	1.014–1.054	<0.001
Living condition	Area of building								
	A big city, the suburbs or outskirts of a big city	8795	3106 (35.3)	1	-	-			
	A large or small town	14,575	5158 (35.4)	0.970	0.915–1.027	0.293			
	A rural area or village	11,722	4195 (35.8)	0.989	0.930–1.051	0.716			
	Type of building								
	A free-standing one/two-family house OR a one- or two-family house as a row or double house	20,502	6999 (34.1)	1	-	-	1	-	-
	A farmhouse	2291	761 (33.2)	0.980	0.891–1.078	0.675	0.837	0.751–0.932	0.001
	A building with 3 or more floors	11,975	4516 (37.7)	1.148	1.092–1.206	<0.001	1.088	1.027–1.153	0.004
	A housing complex with services for older people or a nursing home	324	183 (56.5)	2.678	2.140–3.351	<0.001	1.053	0.812–1.365	0.696

**Table 3:** Comparison of the prevalence of polypharmacy detailed by country, among the 17 countries included in SHARE’s Waves 6 and 9.

	Wave 6 Overall (95% CI)	Wave 9 Overall (95% CI)
Austria	31.5 [30.9–32.1]	31.2 [30.7–31.8]
Germany	30.3 [29.8–30.8]	32.1 [31.5–32.7]
Sweden	31.0 [30.5–31.6]	37.8 [37.2–38.4]
Spain	31.6 [31.0–32.1]	33.8 [33.3–34.4]
Italy	32.9 [32.3–33.5]	32.7 [32.1–33.3]
France	31.0 [30.5–31.6]	28.7 [28.1–29.2]
Denmark	32.7 [32.1–33.2]	39.6 [39.0–40.2]
Greece	29.3 [28.7–29.8]	25.9 [25.4–26.4]
Switzerland	26.3 [25.8–26.8]	26.0 [25.5–26.5]
Belgium	34.0 [33.4–34.6]	38.2 [37.6–38.8]
Israel	37.5 [36.9–38.2]	51.5 [50.8–52.2]
Czech Republic	39.9 [39.3–40.5]	46.6 [45.9–47.3]
Poland	33.8 [33.3–34.4]	51.8 [51.1–52.5]
Luxembourg	29.5 [28.9–30.0]	29.2 [28.6–29.7]
Portugal	36.9 [36.3–37.5]	51.3 [50.6–52.0]
Slovenia	28.1 [27.6–28.6]	25.0 [24.5–25.5]
Estonia	28.4 [27.8–28.9]	33.5 [32.9–34.0]
Croatia	27.3 [26.8–27.9]	32.7 [32.1–33.2]

## Data Availability

The data presented in this study are openly available in http://www.share-project.org/ (SHARE Wave 9—DOI: 10.6103/SHARE.w9.900).

## Supplementary Materials

The following supporting information can be downloaded at: https://www.mdpi.com/article/10.3390/jcm14041330/s1, Detailed Methods.
