# Supplementary material for: Polypharmacy Prevalence Among Older Adults Based on the Survey of Health, Ageing and Retirement in Europe: An Update
Source: J Clin Med. 2025 Feb 17;14(4):1330. doi: 10.3390/jcm14041330 (PMC11856818; doi:10.3390/jcm14041330)
Supplement: Supplementary file 1 [file jcm-14-01330-s001.zip › jcm-3469075-supplementary.docx]

Sociodemographic information included several variables. Gender was recorded as "male" or "female." Age was calculated based on the participants’ reported year of birth relative to 2022 and categorized into three groups: 65–74 years, 75–84 years, and 85+ years. Marital status was assessed through the question, "What is your marital status?", with responses recoded into four categories: "Never married," "Married or in a registered partnership," "Divorced," and "Widowed." Education was evaluated using the question, "How many years have you been in full-time education?", which included receiving tuition, practical work, supervised study, or taking exams. Responses were grouped into four levels: "Low education (0–8 years)," "Moderate education (9–12 years)," "High education (13–16 years)," and "Very high education (17 or more years)." Lastly, Shortage of money was assessed through the question, "How often do you think that shortage of money stops you from doing the things you want to do?" with the response options: "Often," "Sometimes," "Rarely," and "Never."

The behavioural factors included several variables. Ever smoked daily was assessed through the question, "Have you ever smoked cigarettes, cigars, cigarillos, or a pipe daily for at least one year?" with possible responses being "Yes" or "No." Alcohol intake was evaluated using the question, "During the last 7 days, have you had at least one alcoholic beverage?" with the same response options. Engagement in physical activities was divided into two variables: Sports or activities that are vigorous and Activities requiring a moderate level of energy. These were assessed with the questions, "How often do you engage in vigorous physical activity, such as sports, heavy housework, or a job involving physical labour?" and "How often do you engage in activities that require a moderate level of energy, such as gardening, cleaning the car, or going for a walk?" For both, the response options were: "More than once a week," "Once a week," "One to three times a month," and "Hardly ever or never." Additionally, dietary habits were evaluated through the frequency of consumption of four food groups: Dairy products, Legumes or eggs, Meat, fish, or poultry, and Fruits or vegetables. These were assessed using the following questions: "In a regular week, how often do you have a serving of dairy products, such as milk, cheese, yoghurt, or high-protein supplements?", "In a regular week, how often do you have a serving of legumes, beans, or eggs?", "In a regular week, how often do you eat meat, fish, or poultry?", and "In a regular week, how often do you consume a serving of fruits or vegetables?" For all dietary variables, the response options were: "Every day," "3–6 times a week," and "Less than 3 times a week."

Physical functioning was evaluated through three variables. The number of Limitations in Activities of Daily Living (ADLs) was calculated by summing reported difficulties in six tasks: dressing (including shoes and socks), walking across a room, bathing or showering, eating (such as cutting food), getting in or out of bed, and using the toilet (including getting up or down). Similarly, the number of Limitations in Instrumental Activities of Daily Living (iADLs) was derived from the total difficulties in tasks such as using a map in an unfamiliar place, preparing a hot meal, grocery shopping, making telephone calls, taking medications, performing housework or gardening, managing money, leaving the house independently or accessing transportation, and doing personal laundry. Both variables were categorized into three levels: "0," "1," and "2 or more" limitations. Additionally, Limited in Activities Because of Health was assessed using the question "For the past six months at least, to what extent have you been limited because of a health problem in activities people usually do?" with responses categorized as "Severely limited," "Limited, but not severely," and "Not limited."

Physical health was assessed using three variables. The number of Chronic Diseases was determined based on the number of chronic conditions reported by participants and categorized into three groups: "0," "1," and "2 or more." Self-Perception of Health was evaluated through the question "Would you say your health is..." and categorized into "Poor or very poor," "Good," and "Very good or excellent." Lastly, Hospital Stays in the Last 12 Months were assessed with the question "During the last twelve months, have you been in a hospital overnight? Please consider stays in medical, surgical, psychiatric, or any other specialized wards," with responses recorded as "Yes" or "No."

Mental health was evaluated using several variables. Quality of Life and Well-Being were measured through CASP-12, with scores ranging from 12 to 48, where higher scores indicate better quality of life. The Depression Scale (EURO-D) assessed symptoms such as depression, pessimism, suicidal tendencies, guilt, sleep issues, interest, irritability, appetite, fatigue, concentration, pleasure, and crying. Scores range from 0 ("not depressed") to 12 ("highly depressed") and were dichotomized into "with depressive symptoms" (scores ≥4) and "without depressive symptoms" (scores <4). Loneliness was evaluated using the Three-Item Loneliness Scale, with scores ranging from 3 to 9. Responses were recorded into "Without signs of loneliness" (scores 3–5) and "With signs of loneliness" (scores 6–9). Life Satisfaction was measured with the question: "On a scale of 0 to 10, where 0 means completely dissatisfied and 10 means completely satisfied, how satisfied are you with your life?" Lastly, Social Network Satisfaction was assessed through questions regarding satisfaction with personal relationships, using a 0 to 10 scale, where 0 indicates complete dissatisfaction and 10 indicates complete satisfaction.

Living conditions were evaluated using two variables. The first, "Area of Building," was assessed through the question, "How would you describe the area where you live?" Responses were recoded into three categories: "A big city, the suburbs or outskirts of a big city," "A large or small town," and "A rural area or village." The second variable, "Type of Building," was assessed with the question, "Which type of building does the household live in?" Responses were recoded into four categories: "A free-standing one- or two-family house or a one- or two-family house as a row or double house," "A farmhouse," "A building with three or more floors," and "A housing complex with services for older adults or a nursing home."

“Quality of Life and Well-Being”, “Life satisfaction” and “Social Network Satisfaction” were analysed as continuous variables, while the others were considered categorical.
